# Pre-antigenic regulatory signals in osteoarthritis: modulators of dendritic cell activation and joint immune balance

**DOI:** 10.3389/fimmu.2026.1749150

**Published:** 2026-02-06

**Authors:** Jiabao Liang, Andrew Mark Jackson

**Affiliations:** Host Tumour Interactions Group, BioDiscovery Institute, Centre for Cancer Sciences, School of Medicine, University of Nottingham, Nottingham, United Kingdom

**Keywords:** damage-associated molecular patterns, dendritic cells, extracellular matrix stiffness, hypoxia, immuno-regenerative therapy, metabolic stress, osteoarthritis, pre-antigen regulatory signals

## Abstract

Osteoarthritis (OA) is now recognized as an immune-metabolic disorder rather than a simple wear-and-tear disease. Dendritic cells (DCs) in the synovium and subchondral bone link mechanical, biochemical, and metabolic stress to immune imbalance. In the early stage of immune activation, pre-antigenic regulatory signals act before classical antigen presentation and influence how DCs shape joint immunity. Increased extracellular matrix (ECM) stiffness activates the Integrin-FAK-NF-κB pathway, driving inflammatory or exhausted DC states. ECM fragments and damage-associated molecular patterns (DAMPs) stimulate pattern recognition receptors (PRRs), inducing cytokines that sustain chronic inflammation. Hypoxia, lactate, and oxidative stress reprogram DC metabolism, suppress IL-12, and promote Th17 responses. Targeting these upstream factors offers new therapeutic opportunities. Strategies that modify matrix stiffness, block DAMP-mediated signaling, or restore metabolic balance can help reset DC function and preserve joint homeostasis. Emerging biomaterial-based approaches further provide a foundation for immune-restorative and regenerative therapies. In the future, integrating DC-modulatory materials with personalized immune profiling may enable precise immuno-regenerative treatments for OA, representing a shift from symptom relief to immune-guided cartilage repair.

## 1 Introduction

Osteoarthritis (OA) has long been characterized as a wear-and-tear’ condition of the joint, mainly caused by mechanical stress, cartilage breakdown, and changes in subchondral bone (1). However, in recent years, an increasing body of evidence has redefined OA as an immuno-metabolic disorder, where low-grade chronic inflammation and immune-metabolic dysregulation are key factors (2). Growing awareness of the immune system’s role in OA development highlights the crucial functions of resident and infiltrating immune cells in regulating joint inflammation and tissue remodeling.

Immune cells, including DCs, in the synovial cavity, cartilage, and subchondral bone have been increasingly associated with OA pathology. For instance, Li et al. (3) used bulk RNA sequencing and immunohistochemical staining of human OA synovium to demonstrate notable immune cell infiltration, with cytokine profiles correlating with cartilage degradation (3). Similarly, Panichi et al. (4) employed flow cytometry and multiplex cytokine assays to confirm that synovial immune activation parallels matrix breakdown in advanced OA (4). Among these immune populations, DCs serve as early sentinels situated within the synovium and subchondral bone marrow, continually sampling extracellular and metabolic cues (5). As professional antigen-presenting cells, DCs bridge innate immune sensing with adaptive T-cell activation, allowing them to translate subtle environmental stress into distinct immune outcomes. Beyond their traditional role of presenting antigens, DCs also process non-antigenic environmental inputs, positioning them as sensitive transducers of the joint microenvironment.

**Figure 1:**
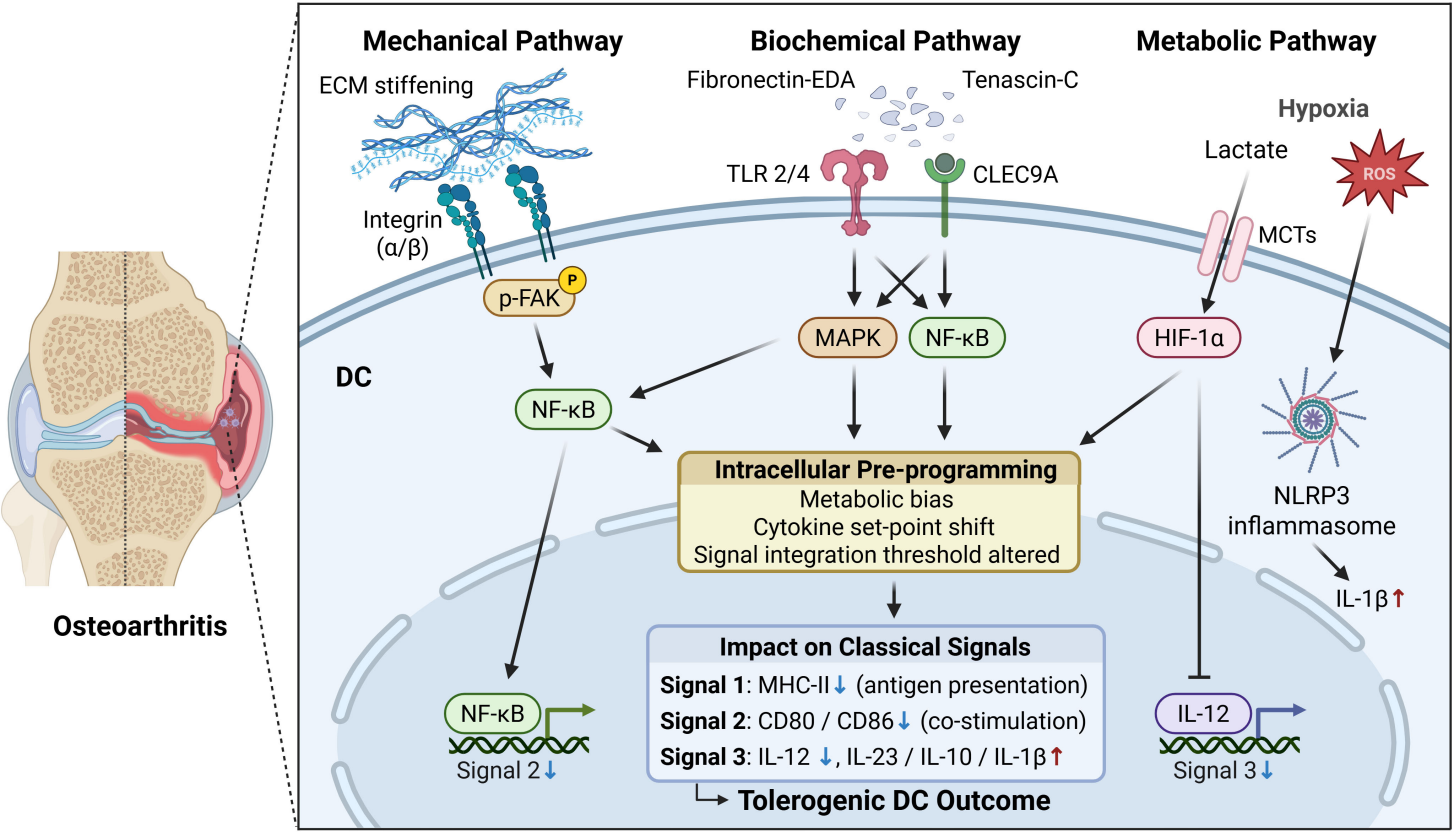
Pre-antigenic regulatory signals linking different factors to DC fate in OA.

Building on this perspective, the concept of early regulatory signals has been introduced to describe upstream cues that occur before the classical antigen-presentation cascade (6), which includes Signal 1 (antigen recognition), Signal 2 (co-stimulation), and Signal 3 (cytokine milieu). Specifically, Signal 1 represents the interaction between peptide–MHC complexes on antigen-presenting cells and T-cell receptors, Signal 2 provides the necessary co-stimulatory engagement through molecules such as CD80/CD86 and CD28, and Signal 3 is mediated by cytokines that determine the direction of T-cell differentiation (7). In this review, pre-antigenic regulatory signals are defined as early, non-antigenic cues that do not form part of Signal 1–3 but influence DC states before antigen presentation occurs. While this concept partially overlaps with the “danger model” and damage-associated molecular patterns (DAMPs)-mediated signaling, it is not equivalent to them (8). Pre-antigenic regulation instead reflects a broader early influence that shapes later immune responses, rather than directly triggering innate immune activation. Within joint tissues, constantly subjected to mechanical loading, biochemical signaling, and metabolic stress, these pre-activation signals are especially relevant (**Figure 1**). DCs detect and process such early stimuli, skewing their polarization toward either an immunogenic or tolerogenic state (9). This modulation consequently influences subsequent T-cell responses and the overall tissue outcome (10). Therefore, this review aims to summarize and discuss recent advances in understanding how such early regulatory signals shape DC fate within the osteoarthritic microenvironment, offering new insight into immuno-metabolic regulation of joint homeostasis and pointing to novel therapeutic targets.

## 2 Mechanical factors: ECM stiffness and joint loading

Extracellular matrix (ECM) stiffening, one of the earliest detectable physical danger signals, initiates the process by which immune cells sense and respond to joint degeneration. Repetitive mechanical loading causes increased collagen cross-linking and changes in ECM, leading to joint tissue stiffening, especially in cartilage, synovium, and subchondral bone. Recent evidence from biomechanical and histological analyses indicates that increased ECM stiffness in OA marks an early sign of mechanical hardening and serves as a crucial factor in irreversible cartilage degeneration (11).

At the cellular level, increased matrix stiffness is sensed through integrin-mediated mechanotransduction, initiating a mechanical signaling cascade in DCs. On stiffer substrates, integrins form more stable clusters and promote focal adhesion assembly, leading to sustained activation of focal adhesion kinase (FAK). Continued FAK signaling engages downstream pathways such as the nuclear factor kappa-light-chain-enhancer of activated B cells (NF-κB) pathway, allowing mechanical inputs to be integrated over time rather than eliciting a transient response (12). As this signaling persists, intracellular programs are gradually reshaped, resulting in altered cytokine set-points, increased matrix metalloproteinase (MMP) expression, and ongoing ECM remodeling, which together reinforce the local mechanical and immune environment (13, 14). Consistent with this model, bioengineering studies further demonstrated that substrates with varying stiffness can direct cell migration, morphology, and signal transduction, confirming the tunable nature of stiffness-dependent mechanotransduction *in vitro* (15).

Within the joint cavity, synovial fibroblasts and DCs are constantly exposed to this dynamic mechanical environment. Increased stiffness and overload trigger synovial fibroblasts to produce more MMPs and inflammatory mediators, leading to ECM remodeling and changes in its immune environment. (16). Meanwhile, DCs can sense substrate stiffness through the Integrin/FAK axis. Under moderate stiffness, DCs may adopt a relatively less inflammatory, more regulated activation program, characterized by altered cytokine secretion and enhanced tissue homeostatic signals (2). Nevertheless, persistent matrix hardening and mechanical overload maintain NF-κB activation in dendritic cells, resulting in the downregulation of co-stimulatory molecules, compromised migration, and functional exhaustion, thus promoting a tolerogenic or immunosuppressive phenotype. In other words, changes in matrix stiffness determine whether DCs follow an activation or tolerance path. Evidence from Panichi et al. (4) suggests that synovial DCs can be mobilized under mechanical stress and take part in chronic low-grade inflammation within OA joints (4).

Early or moderate mechanical stress, such as physiological compression or baseline joint loading, helps preserve joint homeostasis by transiently engaging the integrin-FAK signaling pathway and modulating local immune activity (17). However, persistent or excessive loading continuously stimulates the integrin/FAK-NF-κB axis, leading to sustained MMP release, ECM stiffening, and structural deterioration. Under these conditions, DCs display reduced motility and features of functional exhaustion, shifting toward a tolerogenic phenotype that diminishes T-cell activation and sustains chronic low-grade inflammation (17). Whether early or moderate mechanical stress can transiently activate the FAK pathway to trigger IL-12-driven immune repair and maintain tissue balance remains an open question deserving further investigation.

## 3 Biochemical factors: matrix fragments and DAMPs

The inflammatory nature of osteoarthritis is unique in that it occurs in the absence of infection, defining it as a form of sterile inflammation. This phenomenon challenges the traditional “self-versus non-self” paradigm in immunology, which has historically attributed immune activation to exogenous pathogens (18). In the late 1990s, Polly Matzinger introduced the “danger model”, proposing that the immune system can also be triggered by endogenous molecules released from stressed or damaged cells (8). These endogenous alarm signals, subsequently known as DAMPs, serve to bridge the gap between tissue injury and immune activation.

In the osteoarthritic joint microenvironment, continuous mechanical overload, oxidative stress, and ECM degradation convert otherwise inert self-molecules into immunostimulatory DAMPs. These molecules are now recognized not merely as by-products of cartilage degeneration but as active mediators that sustain immune imbalance and chronic inflammation. Through proteomic and cellular analyses, Dang et al. (19) demonstrated that ECM fragments and cartilage debris recruit and activate DCs and macrophages in the synovium, amplifying local inflammatory loops (19).

Among ECM-derived DAMPs, fibronectin-EDA, biglycan, and tenascin-C are considered key mediators linking tissue degradation to immune activation. These matrix fragments can bind to multiple pattern recognition receptors (PRRs) on the surface of DCs, primarily including Toll-like receptors (TLRs) and C-type lectin receptors (CLRs). Fibronectin-EDA and biglycan primarily interact with TLR2 and TLR4, activating the NF-κB and MAPK cascades, which in turn induce the expression of IL-23, IL-6, TNF-α, and costimulatory molecules such as CD80 and CD86 (20, 21). In contrast, tenascin-C and certain ECM fragments released from necrotic cell debris are recognized by C-type lectin domain family 9 member A (CLEC9A), leading to IL-1β production and the regulation of antigen cross-presentation (22).

DAMP signaling affects DCs in a biphasic manner: brief or weak stimulation drives DCs toward a pro-inflammatory state, characterized by increased IL-23 and IL-6 levels, which promotes T helper 17 cell (Th17) expansion and perpetuates inflammation (23). In contrast, prolonged or excessive exposure to DAMPs fosters a tolerogenic state characterized by increased IL-10 expression, decreased co-stimulatory molecules (such as CD80 and CD86), and a diminished capacity for T-cell activation (24). This plasticity, from activation to tolerance, positions DCs as both triggers of inflammation and players in immune exhaustion within chronic OA (19, 24).

## 4 Metabolic factors: hypoxia, lactate, and oxidative stress

A hypoxic microenvironment is a defining feature of osteoarthritic joints, playing a crucial role in immune and metabolic imbalances. Sustained oxygen deprivation stabilizes hypoxia-inducible factor-1α (HIF-1α) in DCs, leading to a metabolic shift toward glycolysis while inhibiting IL-12 production and Th1 priming (25). This change alters the cytokine profile and reduces DC migration and antigen presentation, additionally disturbing immune homeostasis in the joint (26, 27). Recent work has shown that prolonged hypoxia in OA cartilage and synovium also reprograms chondrocyte metabolism, increasing lactate and reactive oxygen species (ROS) production, thereby reinforcing a cycle of matrix degradation and immune activation (28).

At the same time, lactate accumulation represents another hallmark of metabolic disturbance in OA. Notably, sustained lactate exposure acts as a time-dependent signal, not a one-off trigger. As cartilage breaks down and synovial cells shift toward glycolysis, intra-articular lactate rises and local pH declines. Immune cells take up lactate mainly through monocarboxylate transporters (MCTs) and also sense extracellular lactate via hydroxycarboxylic acid receptors (HCARs) (29). In parallel, acidosis is detected by G protein-coupled receptors (GPCRs), which further shape intracellular signaling and immune tone in inflamed tissues (30, 31). Persistent exposure to elevated lactate gradually reprograms DC metabolic preferences and signaling thresholds, resulting in reduced antigen presentation capacity and a shift in cytokine output toward IL-23 and IL-6, which favors Th17-skewed inflammation (32). In this context, lactate functions not merely as a metabolic by-product, but as a sustained regulatory signal that biases DC functional states under chronic inflammatory conditions (33).

ROS further amplify this metabolic network. Excess ROS in chondrocytes and synovial fibroblasts activate the NOD-like receptor family pyrin domain-containing 3 (NLRP3) inflammasome, leading to the release of Interleukin-1β (IL-1β) and IL-18 and accelerating cartilage matrix degradation (34). In OA, the ROS-NLRP3 pathway is gradually recognized as a critical driver of sterile inflammation and tissue degeneration (35). Beyond inflammasome activation, persistent oxidative stress disrupts intracellular redox balance and mitochondrial function in DCs, progressively limiting antigen-processing capacity and immune flexibility (36, 37). Together, hypoxia, lactate, and ROS act in concert to shift DCs from an active immune state to a tolerant one, characterized by reduced IL-12 and increased IL-23 and IL-6 production (28). This imbalance helps sustain the persistent, low-grade inflammation typical of osteoarthritic joints.

## 5 Discussion and therapies

**Figure 2:**
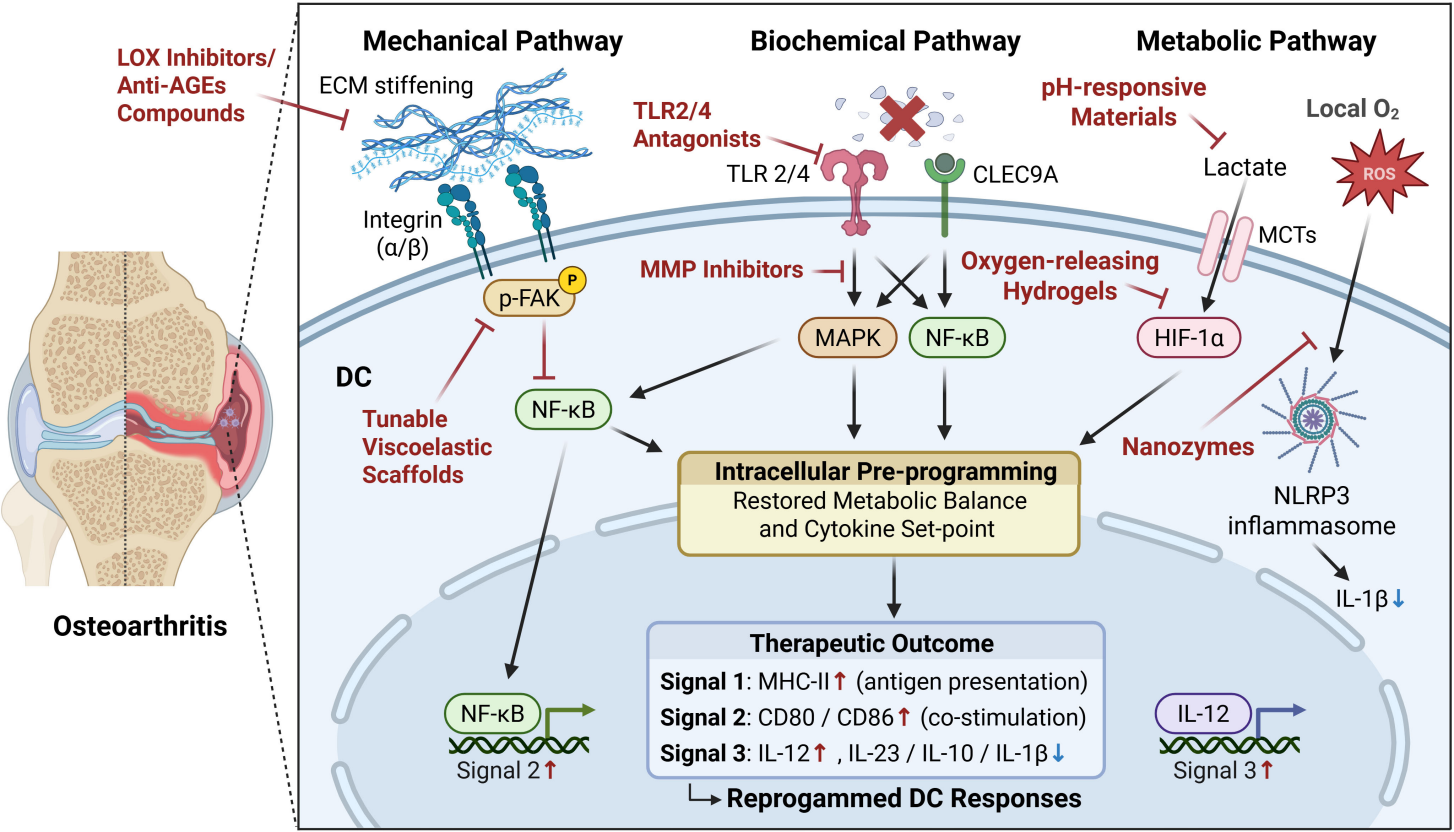
Therapeutic strategies targeting pre-antigenic signals to restore DC function in OA.

The previous section discussed how mechanical, biochemical, and metabolic cues collectively shape DC fate in OA. When these pre-antigenic cues persist within the OA joint, they progressively disrupt the downstream activation cascade of DCs, shifting them from an immunogenic toward a tolerogenic state (**Figure 1**). Major Histocompatibility Complex class II (MHC II) expression decreases, antigen-loading efficiency is reduced, and the ability of DCs to form stable immunological synapses with T cells is weakened (Signal 1); costimulatory molecules such as CD80, CD86, and CD40 are downregulated or functionally impaired, leading to insufficient T-cell priming (Signal 2); meanwhile, the cytokine milieu adopts a regulatory phenotype (Signal 3), with increased IL-10 and decreased IL-12 and IL-6 production (38). As a result, effector T-cell activation is attenuated, regulatory T-cell (Treg) differentiation is promoted, and both immune clearance and reparative responses remain chronically suppressed, sustaining the low-grade inflammation characteristic of OA (24). Given this downstream impairment, reprogramming the joint immune microenvironment offers two potential therapeutic avenues: modulating the upstream cues themselves or directly guiding DC differentiation (**Figure 2**).

At the level of signal regulation, several innovative strategies have emerged. Inhibition of lysyl oxidase (LOX) activity or the formation of advanced glycation end products (AGEs), combined with the use of scaffolds with tunable viscoelasticity, has been shown to relieve matrix stiffening and mechanical stress in osteoarthritic cartilage (11, 39, 40). Biochemical danger signals, such as DAMP-induced activation of TLR2/4 and MMP-driven matrix degradation, may be attenuated using TLR antagonists (41, 42) or MMP inhibitors (43), both of which help to maintain extracellular matrix integrity and limit local inflammation (44).

Metabolic stress can also be targeted through material-based approaches. Oxygen-releasing hydrogels have recently been developed to elevate oxygen tension in hypoxic cartilage, restore redox equilibrium, and promote cartilage repair, thereby lifting DCs from metabolically restricted states that limit their immunogenicity (45–47). Similarly, pH-responsive nanomaterials and hydrogel–nanoparticle composite systems can buffer acidic environments or eliminate excess lactate, which helps to normalize immune cell function and preserve joint homeostasis (48–50). In addition, manganese-based nanozymes and other antioxidant nanocatalysts have demonstrated potent scavenging activity against ROS, thereby mitigating oxidative stress and slowing OA progression (51, 52). By restoring the microenvironment from a pro-degenerative to a balanced state, the intrinsic reparative capacity of DCs may be reactivated, contributing to tissue regeneration.

Beyond manipulating upstream cues, a complementary strategy focuses on directing DCs toward pro-repair phenotypes. Mature DCs that express high levels of IL-12 favor Th1-dominant responses linked to controlled repair, whereas immature or tolerogenic DCs with increased IL-23 or IL-10 production promote Th17-driven inflammation and cartilage degradation (53–55). Recent advances in nanotechnology enable the precise delivery of cytokine modulators or small-molecule inhibitors to DCs, allowing fine control over their maturation and function within the synovial microenvironment (48–50, 56, 57). Single-cell transcriptomic analyses have further identified DC-like subsets associated with OA progression, providing new evidence for the feasibility of DC-targeted immune interventions (58–60).

## 6 Limitations and challenges

While the concept of pre-antigenic regulatory signals offers a helpful framework for interpreting DC reprogramming in OA, several limitations need to be recognized. The line between pre-antigenic regulation and traditional innate immune activation is inherently blurry, as these processes likely exist on a spectrum and share key signaling pathways such as NF-κB, HIF-1α, and inflammasome-related pathways, making precise timing difficult to determine *in vivo* (2, 28). In addition, much of the current evidence is derived from reductionist models that isolate mechanical, metabolic, or biochemical cues and often capture acute responses, rather than the chronic and combinatorial stresses that characterize the osteoarthritic joint (11, 26). Moreover, the marked heterogeneity of synovial DC subsets further complicates translation, as broadly targeting upstream signals may unintentionally disrupt protective immune functions or tissue repair programs revealed by single-cell studies (5, 59). Long-term modulation of DC activation or joint metabolism may carry safety concerns, including impaired local immune surveillance or unintended systemic effects, highlighting the need for tightly controlled therapeutic strategies (25, 28).

## 7 Conclusion and outlook

Taken together, precise pre-antigenic control of mechanical, biochemical, and metabolic cues is essential for shaping DC behavior in OA. When these early signals are properly balanced, DCs can support tissue protection and repair rather than driving inflammation. By guiding DCs toward repair-oriented states, it may be possible to slow or even reverse the course of OA instead of only relieving symptoms. Future research should aim to better understand how these signals interact over time and how different levels of stress influence DC function in the joint. Combining laboratory models with patient-derived data could help identify the key stages where intervention is most effective. In treatment design, soft and adaptive biomaterials that mimic the natural joint environment, together with DC-targeted therapies, may help restore immune balance and promote cartilage recovery. The integration of these materials with personalized immune profiling could eventually make it possible to design patient-specific immuno-regenerative treatments, marking a major step from symptomatic care to long-term immune-guided repair.
